# Reproductive performance and its risk factors in Hanwoo cattle: a large-scale field study

**DOI:** 10.5713/ab.260249

**Published:** 2026-06-01

**Authors:** Hyun-Gu Kang, Jae-Kwan Jeong, Ill-Hwa Kim

**Affiliations:** 1 College of Veterinary Medicine, Chungbuk National University, Cheongju, Korea; 2 Warm Vet Clinic, Cheongju, Korea

**Keywords:** Breeding Season, Hanwoo Cattle, Parity, Reproductive Performance, Risk Factors, Synchronization Protocol

## Abstract

**Objective:**

This study aimed to identify factors affecting reproductive performance in Hanwoo cattle.

**Methods:**

A total of 4,656 artificial insemination (AI) records were analyzed to evaluate factors associated with pregnancy per AI, pregnancy loss, the number of inseminations per conception, and the time to pregnancy.

**Results:**

The probability of pregnancy per AI was lower in cattle inseminated during spring than in those inseminated during winter, and lower in heifers than in cows. In contrast, pregnancy probability was higher in cattle with synchronization protocols including PGF_2α_-GnRH, modified Double-Ovsynch, and modified Presynch-Ovsynch, compared with insemination after detected estrus. The risk of early pregnancy loss between 31 and 59 days of gestation was higher following the first insemination than after the second, while the risk of late pregnancy loss between 60 and 260 days was higher in heifers than in cows. The number of inseminations per conception was higher in heifers and in cattle experiencing early or late pregnancy loss. The implementation of modified Double-Ovsynch and modified Presynch-Ovsynch during the first insemination was associated with fewer inseminations compared to AI after detected estrus. The probability of pregnancy by 360 days postpartum was reduced in cows with intervals from calving to first insemination of 61 to 80 days or 81 days or more, compared with those inseminated within 60 days, as well as in cows that experienced early or late pregnancy loss. Conversely, the likelihood was higher in cows that calved during spring than those that calved during winter.

**Conclusion:**

Heifer status, insemination during spring, and pregnancy loss adversely affected reproductive performance in Hanwoo cattle. In contrast, the use of synchronization protocols, earlier postpartum insemination, and spring calving were associated with improved reproductive outcomes. These findings support the necessity for targeted interventions to improve reproductive efficiency in Hanwoo herds.

## INTRODUCTION

Hanwoo (Korean native cattle, *Bos taurus coreanae*) is the primary beef breed in Korea and a major contributor to beef production in the country. As with other cattle breeds, improvements in reproductive performance have a substantial impact on farm profitability in Hanwoo herds. Achieving optimal reproductive efficiency is therefore essential and includes maintaining a 12-month calving interval, increasing pregnancy rate per artificial insemination (AI), and reducing pregnancy loss and the number of inseminations per conception [1,2].

In Korea, the reproductive efficiency of Hanwoo cattle is compromised by poor reproductive management, mainly due to unfavorable conditions for feed production, crowding in feedlots, and the continuous expansion of farm size under an intensive production system. Consequently, inadequate nutritional status, poor estrus detection, and increased postpartum anestrus have been reported as major factors negatively affecting reproductive performance in Hanwoo herds [3,4]. Several studies have sought to improve reproductive performance in Hanwoo cattle through nutritional management, automated estrus monitoring, and the application of synchronization protocols [3,5,6]. Regular reproductive check-ups also enhanced reproductive efficiency by assessing individual health and reproductive disorders, diagnosing early pregnancy using ultrasonography, and implementing synchronization protocols in herds [7]. Nevertheless, there remains a lack of comprehensive field-based reproductive technologies and information to improve reproductive efficiency in Korean beef herds. We therefore hypothesized that identifying factors affecting reproductive performance under domestic reproductive management and environmental conditions would broaden understanding of the causes of declining farm fertility. Applying this information at the farm level could improve reproductive outcomes.

Compared with dairy herds, relatively few studies have investigated factors influencing reproductive performance in beef herds. This may reflect the perception that reproductive efficiency has a smaller economic impact in beef production than in dairy systems and the limited accumulation of large-scale data on factors affecting reproductive efficiency in beef cattle. However, identifying associated risk factors is essential in beef herds, particularly in regions with intensive reproductive management [8]. Previous studies have reported that age, breed, body condition score (BCS), health status, bull infertility, breeding season, and the use of synchronization protocols influence reproductive performance in beef cattle [8–10]. These effects may vary across regions, production systems, management practices, breeds, and animal characteristics. In Korea, few studies have used large-scale data to examine risk factors for reproductive efficiency in Hanwoo cattle. Therefore, this study aimed to identify factors affecting reproductive performance in Hanwoo cattle using large-scale reproductive data collected during routine farm visits, with the goal of informing strategies to improve reproductive efficiency in Hanwoo herds.

## MATERIALS AND METHODS

### Hanwoo herds

This study was conducted on 34 Hanwoo farms located in Chungcheong Province, Korea. Each farm housed between 30 and 130 Hanwoo cattle. The farm size distribution was 64.7% for farms with 59 or fewer cattle and 35.3% for farms with 60 or more cattle. All cattle were maintained in indoor feedlots and fed a diet consisting of concentrate, rice straw, minerals, and vitamins. Drinking water was provided *ad libitum*. Feedlots were equipped with fans and shade during the hot months from June to September.

### Reproductive management

Monthly reproductive health examinations were performed on all cattle by the same veterinarians from the research team. Examinations included ultrasonographic evaluation of ovarian structures and the uterus using a 6.5 MHz array transducer (SR-1C-Wireless Rectal Scanner; SongKang GLC). Calves were weaned at an average of 3 months of age. Heifers received their first AI at approximately 330 days of age, following estrus detection. Cows received their first AI approximately 40 days after calving, following detection of estrus.

In addition to AI following detected estrus, five synchronization protocols were applied for the first and subsequent AIs in heifers and cows as follows: (1) administration of 500 μg of a prostaglandin F_2α_ (PGF_2α_) analog, cloprostenol sodium (Estrumate; MSD Animal Health), followed by an injection of 10 μg of a gonadotropin-releasing hormone (GnRH) analog, buserelin acetate (Gestar, Over), with timed AI (TAI) 72 h after PGF_2α_ injection (PGF_2α_-GnRH); (2) injection of GnRH, followed by PGF_2α_ 7 d later, a second dose of GnRH 56 h after PGF_2α_ injection, and TAI 16 h after the second GnRH injection (Ovsynch, [11]); (3) injection of GnRH, followed by the Ovsynch protocol 6 days later (mean±standard deviation [SD], 6±1.5 days) (GnRH-Ovsynch); (4) injection of GnRH, followed by PGF_2α_ 11 days later (mean±SD, 11±1.1 days), GnRH 3 days after the first PGF_2α_ injection, and then the Ovsynch protocol 7 days later (modified Double-Ovsynch); and (5) injection of PGF_2α_, followed by a second dose of PGF_2α_ 11 days later (mean±SD, 11±1.1 days), GnRH 3 days after the second PGF_2α_ injection, and then the Ovsynch protocol 7 days later (modified Presynch-Ovsynch). The PGF_2α_-GnRH protocol was applied only to cattle with a functional corpus luteum (CL) measuring larger than 15 mm in diameter and appearing hypoechoic on ultrasonography.

Pregnancy diagnosis was performed by transrectal ultrasonography using a 6.5 MHz array transducer at approximately 31 days (mean±SD, 31±3.4 days) and 59 days (mean±SD, 59±10.0 days) after AI. A heifer or cow was considered pregnant at 31 days if a CL was present and a viable embryo with a heartbeat and clearly defined anechoic amniotic fluid was observed in the uterus. Pregnancy at 59 days was confirmed by the presence of a living fetus with normal fetal movements. A pregnancy confirmed at 31 days but absent at 59 days was defined as early pregnancy loss (late embryonic or early fetal loss). A pregnancy confirmed at 59 days post-AI but subsequently lost, as indicated by observed estrus or by detection of an aborted or mummified fetus by 260 days post-AI, was defined as late pregnancy loss (mid to late fetal loss). Thus, pregnancy loss was categorized as early or late using a cutoff at approximately 60 days post-AI, corresponding to the time when implantation is firmly established, and the risk of loss is substantially reduced; this distinction was used to differentiate stage-specific risk factors [12].

Cattle that failed to conceive after the first AI and subsequently exhibited estrus were inseminated according to the am-pm rule. When cattle were confirmed non-pregnant, the synchronization protocols described above were repeated until pregnancy was achieved or the animal was culled.

### Data collection and study design

This study was conducted on 34 Hanwoo farms located in Chungcheong Province, Korea. A total of 4,656 AI records from 1,766 Hanwoo cattle (757 heifers and 1,009 cows) were collected between 2015 and 2024. During the study period, the monthly mean ambient temperature ranged from −1.48°C in January to 26.0°C in August, and the relative humidity ranged from 69.7% to 87.1% across seasons. The dataset included parity, synchronization protocols, herd size, and dates of reproductive events, including calving, AI, pregnancy diagnosis, and pregnancy loss.

The following reproductive parameters were evaluated in this study: the probability of pregnancy per AI, the prevalence of early and late pregnancy loss, the number of inseminations required per conception, and the probability of pregnancy by 360 days postpartum. Findings for these parameters were integrated to guide the development of strategies to improve reproductive efficiency in Hanwoo herds.

### Statistical analyses

Results are expressed as means±standard error of the mean, except where descriptive statistics are expressed as means±SD. Statistical analyses were performed using SAS statistical software (ver. 9.4; SAS Institute). For analyses, cattle were categorized by parity (heifer and cow); AI type (insemination after detected estrus or synchronization protocols, including PGF_2α_-GnRH, Ovsynch, GnRH-Ovsynch, modified Double-Ovsynch, and modified Presynch-Ovsynch); AI number (first, second, or ≥third); herd size (<60 or ≥60 cattle); and interval between calving and first AI (≤60, 61 to 80, or ≥81 days). The seasons of calving and AI were defined as spring (March to May), summer (June to August), autumn (September to November), and winter (December to February). Pregnancy loss was classified as early (31 to 59 days of gestation) or late (60 to 260 days of gestation).

The overall probability of pregnancy per AI and the probability of pregnancy loss during early and late gestation were analyzed using logistic regression with the LOGISTIC procedure. The models included season of AI, parity, AI type, AI number, herd size, and interactions among these variables. Backward stepwise regression was applied, with variables removed when p>0.1 based on the Wald statistic. Odds ratios (OR) and 95% confidence intervals were calculated.

The number of inseminations per conception was analyzed using a general linear model that included parity, pregnancy loss category (no loss, early loss, or late loss), AI type at first AI, and herd size as explanatory variables. The probability of pregnancy by 360 days postpartum was analyzed using a Cox proportional hazards model with the PHREG procedure. We selected the 360-day cutoff because in our production system, cows that remain open at or beyond approximately 360 days postpartum are typically classified as infertile and considered for involuntary culling. The Cox model yielded an estimate of the probability of a cow being pregnant at a given time; the time variable was defined as the interval in days between calving and conception. The Cox model included the interval between calving and first AI, pregnancy loss category, season of calving, AI type at first AI, herd size, and interactions among these variables. Proportional hazards were assessed using interactions between explanatory variables and time, and by evaluating Kaplan-Meier curves. Mean intervals between calving and conception were determined using the Kaplan-Meier method in the LIFETEST procedure. A survival plot was generated using the Survival option in MedCalc Software (ver. 11.1; MedCalc Software). For both analyses, cows that died, were sold before conception, or were not pregnant by 360 days postpartum were excluded. The distribution of records across categories of each model effect, stratified by parity where applicable, is presented in Supplements 1–4. A p- value<0.05 was considered significant, and 0.05≤p<0.1 was considered to indicate a tendency toward a significant difference.

## RESULTS

### Overall reproductive performance of Hanwoo cattle

Table 1 summarizes the overall reproductive performance of Hanwoo cattle and compares it with previous reports [13–19]. The overall mean ages at first AI and first calving in heifers were 14.6 months (438.0 days) and 25.2 months (757.3 days), respectively. The overall mean intervals from calving to first AI and to pregnancy in Hanwoo cows were 68.8 days and 91.2 days, respectively. Pregnancy per AI at 59 days, across first and subsequent AIs, was 60.5%. The overall mean number of inseminations per conception was 1.53. The prevalence of early pregnancy loss (31 to 59 days of gestation) and late pregnancy loss (60 to 260 days of gestation) was 1.9% (54/2,872) and 2.2% (63/2,872), respectively, resulting in a total pregnancy loss rate of 4.1%.

### Factors affecting the probability of pregnancy per artificial insemination

Table 2 shows the factors affecting the probability of pregnancy per AI. AI performed during spring was associated with a lower probability of pregnancy than AI performed during winter (OR: 0.76). Heifers had a lower probability of pregnancy than cows (OR: 0.84). In contrast, cows subjected to synchronization protocols, including PGF_2α_-GnRH (OR: 1.19), modified Double-Ovsynch (OR: 1.21), and modified Presynch-Ovsynch (OR: 1.58), were more likely to become pregnant than cows inseminated after detected estrus. Pregnancy probability tended to be higher after the second AI than after the first.

### Factors affecting the prevalence of pregnancy loss following artificial insemination

Table 3 presents the factors affecting pregnancy loss following AI. The probability of early pregnancy loss (31 to 59 days of gestation) was lower after the second AI than after the first AI (OR: 0.30). However, heifers had a higher probability of late pregnancy loss (60 to 260 days of gestation) than cows (OR: 1.82).

### Factors affecting the number of inseminations per conception

Table 4 summarizes the factors affecting the number of inseminations per conception. The least squares mean number of inseminations was significantly higher in heifers than in cows (p<0.0001). Cattle that experienced early or late pregnancy loss required a greater number of inseminations compared with those without pregnancy loss (p<0.0001). In contrast, cattle that received synchronization protocols for the first postpartum AI, including modified Double-Ovsynch (p<0.05) or modified Presynch-Ovsynch (p<0.001), required fewer inseminations per conception than cattle inseminated after detected estrus, whereas PGF_2α_-GnRH showed a nonsignificant trend (p<0.1).

### Factors affecting the likelihood of pregnancy by 360 days postpartum

**Figure 1:**
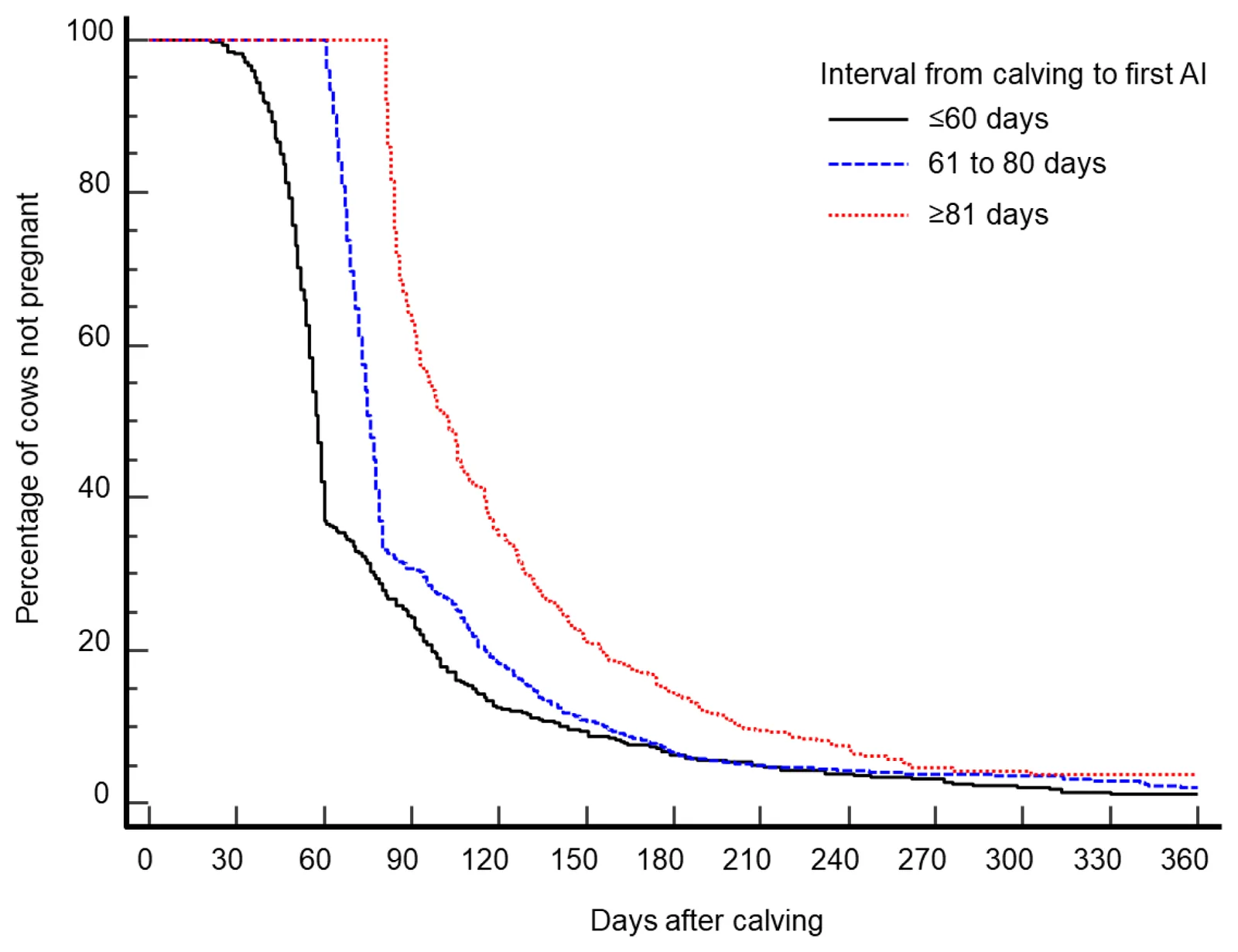
Survival curves show the interval between calving and pregnancy in cows inseminated ≤60 d (solid black line, n = 645), 61 to 80 d (dashed blue line, n = 1,093), or ≥81 d (dotted red line, n = 406) after calving; curves were generated using MedCalc software. The probability of pregnancy by 360 d postpartum was lower (p<0.0001) in cows inseminated 61 to 80 d after calving (hazard ratio [HR]: 0.64) and in cows inseminated ≥81 d after calving (HR: 0.38), compared with cows inseminated ≤60 d after calving. The mean interval from calving to pregnancy (mean±SD) was 79.5±2.4 d, 98.5±1.8 d, and 127.3±3.3 d for the ≤60 d, 61 to 80 d, or ≥81 d groups, respectively (p<0.0001). SD, standard deviation.

**Figure 2:**
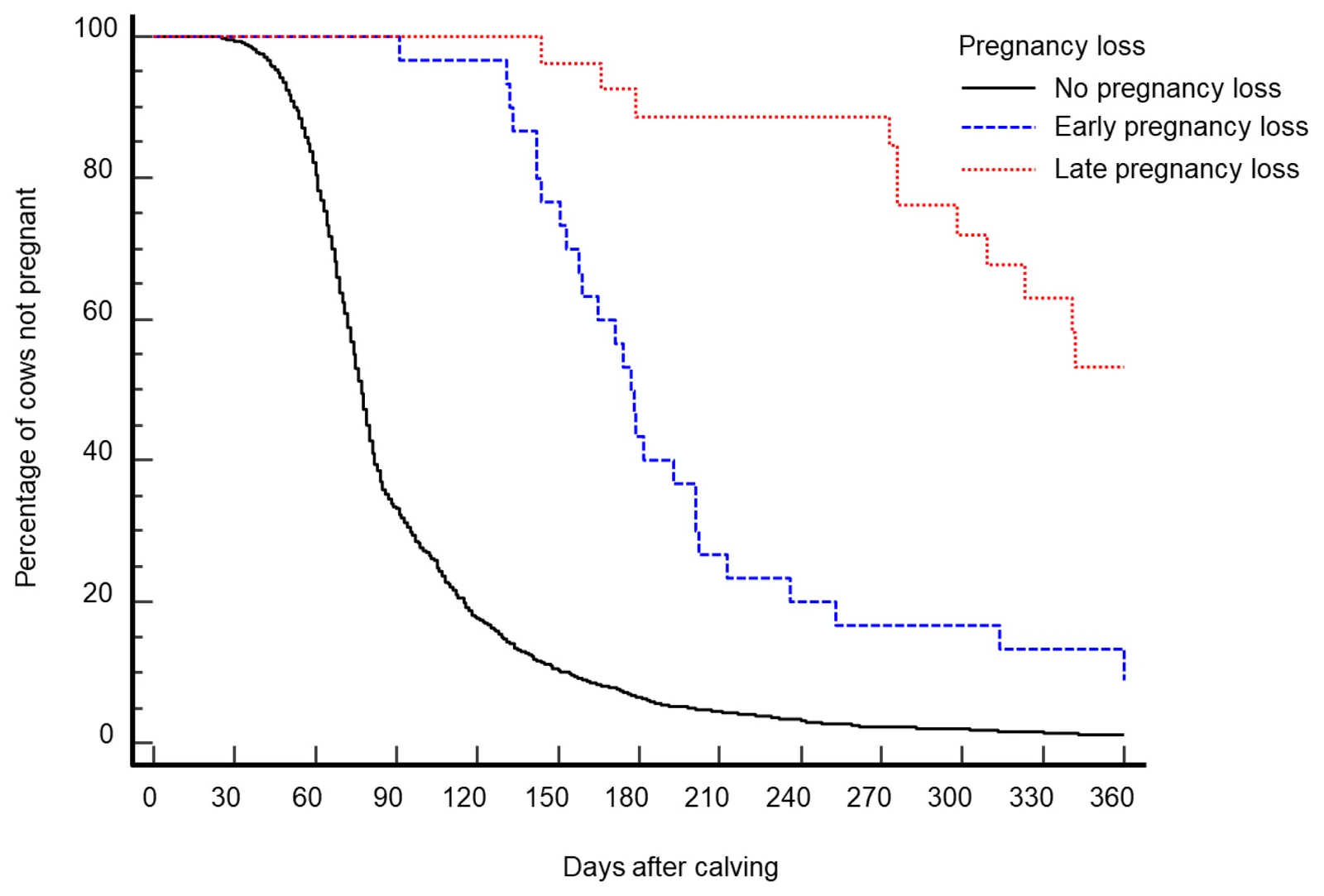
Survival curves generated using MedCalc software show the interval between calving and pregnancy in Hanwoo cows: without pregnancy loss (solid black line, n = 2,087); with early pregnancy loss from 31 to 59 d of gestation (dashed blue line, n = 30); and with late pregnancy loss from 60 to 260 d of gestation (dotted red line, n = 27). The probability of pregnancy by 360 d postpartum was lower (p<0.0001) in cows with early pregnancy loss (hazard ratio [HR]: 0.33) and in cows with late pregnancy loss (HR: 0.11), compared with cows without pregnancy loss. The mean interval from calving to pregnancy was 93.6±1.2 d in cows without pregnancy loss, 200.5±31.9 d in cows with early pregnancy loss, and 318.8±12.4 d in cows with late pregnancy loss (mean±SD; p<0.0001). SD, standard deviation.

**Figure 3:**
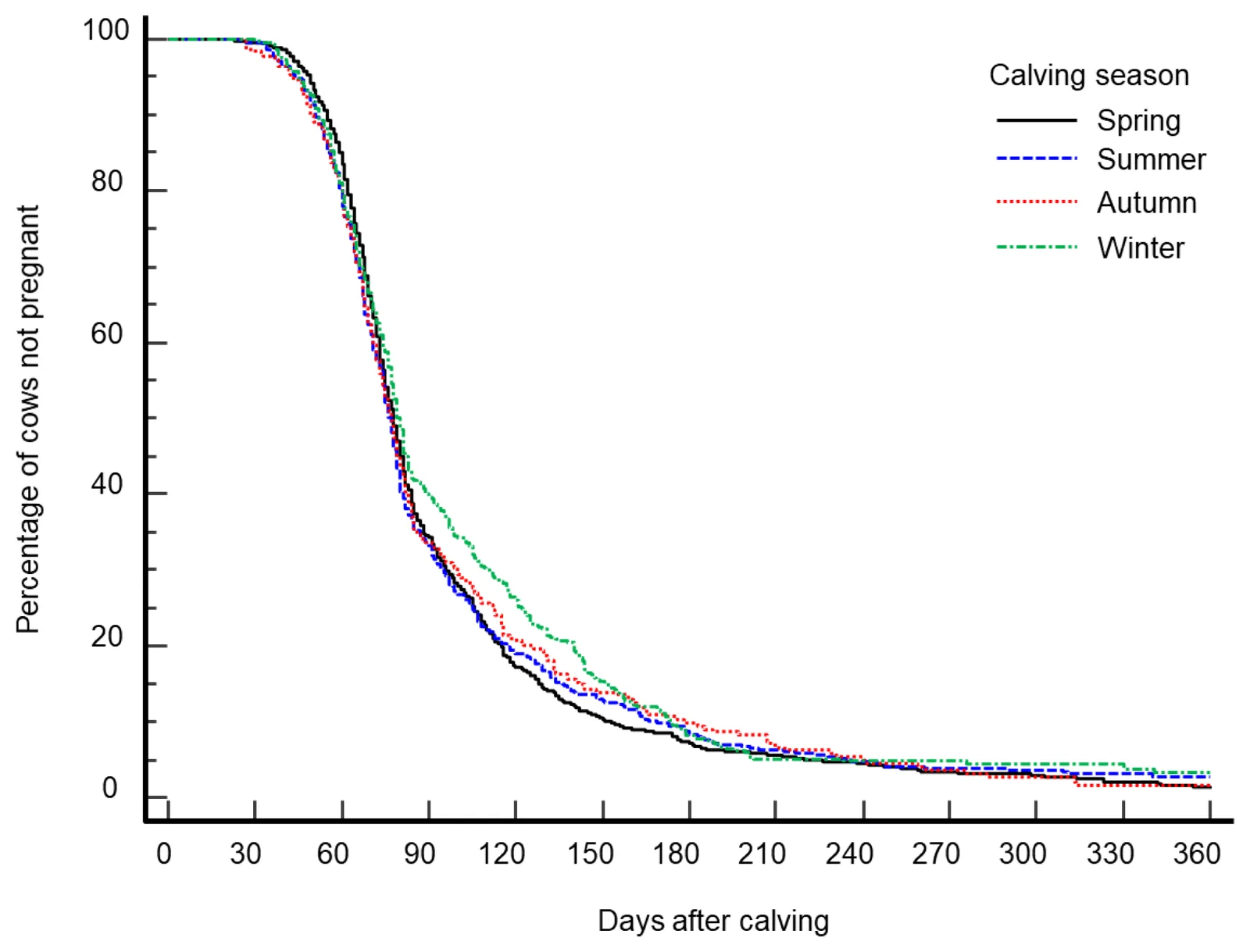
Survival curves show the interval between calving and pregnancy in cows that calved during spring (solid black line, n = 921), summer (dashed blue line, n = 515), autumn (dotted red line, n = 300), or winter (dash dot green line, n = 408); curves were generated using MedCalc software. The probability of pregnancy by 360 d postpartum was higher in cows that calved during spring than in cows that calved during winter (hazard ratio [HR]: 1.13, p<0.05), whereas cows that calved during summer or autumn did not differ from winter-calving cows (p>0.1). The mean interval from calving to pregnancy was 96.4±2.0 d in spring-calving cows and 103.8±3.4 d in winter-calving cows (mean±SD; p<0.05). SD, standard deviation.

Table 5 presents the factors influencing the likelihood of pregnancy within 360 days postpartum following AI. Cows with intervals from calving to first AI of 61 to 80 days (hazard ratio [HR]: 0.60) or 81 days or more (HR: 0.34) were less likely to become pregnant than cows inseminated within 60 days. These delays extended the mean calving-to-pregnancy interval by 19.0 and 47.8 days, respectively (p<0.0001, Figure 1). Cows that experienced early (HR: 0.28) or late (HR: 0.08) pregnancy loss were also less likely to conceive, resulting in extensions of 106.9 and 225.2 days in the calving-to-pregnancy interval, respectively (p<0.0001, Figure 2). In contrast, cows that calved during spring were more likely to become pregnant than those that calved during winter (HR: 1.15), with a shorter mean calving-to-pregnancy interval by 7.4 days (p<0.05, Figure 3).

## DISCUSSION

This study evaluated factors influencing the reproductive performance of Hanwoo cattle and demonstrated that heifer status, AI during spring, and a history of early or late pregnancy loss were associated with poorer reproductive outcomes. In contrast, the use of three synchronization protocols, early postpartum breeding, and spring calving were associated with improved reproductive performance.

The overall reproductive performance observed in the present study was comparable to that reported in earlier Korean studies [13–19], indicating that our study population may be broadly similar to Hanwoo herds managed under typical conditions in Korea.

The probability of pregnancy per AI was significantly influenced by AI season, parity, and the use of synchronization protocols. Cattle inseminated in spring had lower pregnancy rates than those inseminated in winter, which may reflect nutritional and metabolic challenges at the time of insemination. Most cattle (80%) inseminated in spring had calved in winter and therefore may have experienced cold stress and negative energy balance during the transition period, which could have reduced fertility. Notably, cattle inseminated in summer did not differ significantly from those inseminated in winter, contrary to our expectations, because heat stress often reduces pregnancy rates. Possible explanations include effective on-farm measures to mitigate heat stress and the relative heat tolerance of Hanwoo cattle as a beef breed adapted to local conditions. Although similar seasonal effects have been reported in other studies [1,9], one study found different results [8], suggesting that the impact of AI season on fertility is complex and depends on climatic conditions, regional characteristics, breed differences, and management practices. Parity also affected pregnancy per AI: heifers had a lower probability of pregnancy than cows, consistent with previous reports [1,20]. This likely reflects differences in nutritional status, reproductive maturity, estrus expression, AI timing, or responsiveness to synchronization protocols [21–23], indicating that replacement heifers and mature cows differ in reproductive physiology and management needs [24].

Cattle treated with PGF_2α_-GnRH, modified Double-Ovsynch, or modified Presynch-Ovsynch protocols had a higher probability of pregnancy than those inseminated after detected estrus. In contrast, pregnancy outcomes in cattle subjected to Ovsynch or GnRH-Ovsynch protocols were similar to those of cattle inseminated after estrus detection. The precise reasons for these differences are difficult to determine from the present dataset. However, it is plausible that cows that had not resumed cyclicality prior to protocol initiation, or that failed to ovulate a dominant follicle after the first GnRH administration, may have experienced reduced pregnancy rates owing to failure of timed ovulation, as previously reported [25]. The PGF_2α_-GnRH protocol probably improved outcomes through a different mechanism: it was applied only to cattle with confirmed functional luteal activity. Furthermore, the modified Double-Ovsynch and modified Presynch-Ovsynch protocols may have improved synchronization and ovulatory response through more precise hormonal control, as demonstrated in previous studies [26,27]. These findings suggest that such protocols can be effectively applied in clinical practice. Specifically, modified Double-Ovsynch and modified Presynch-Ovsynch protocols may be useful for replacement heifers during the early breeding period or for postpartum cows during the voluntary waiting period, even in the absence of functional CL, thereby improving pregnancy outcomes. In contrast, the PGF_2α_-GnRH protocol may be a cost-effective option for heifers or cows with a functional CL that require breeding within a short time frame. Despite extensive research and field application in dairy cattle [28,29], the use of presynchronization-Ovsynch protocols, such as Presyn-Ovsynch or Double-Ovsynch, in beef herds remains limited [7,30], highlighting the practical relevance of the present findings.

Regarding pregnancy loss, the probability of early pregnancy loss between 31 and 59 days of gestation was higher after the first AI than after the second AI, which may be related to incomplete uterine involution, energy deficiency from negative energy balance, and suboptimal luteal function around the first AI [31,32]. Nutritional status, health, and reproductive recovery may improve with longer postpartum intervals [33,34]. These mechanisms may explain why pregnancy probability tended to be higher after the second AI than after the first. Pregnancy loss after the third AI did not differ from that following the first AI; the mechanism remains unclear but may reflect underlying compromised fertility. Conversely, the probability of late pregnancy loss between 60 and 260 days of gestation was higher in heifers than in cows, consistent with previous findings [12], and may be related to immature reproductive development and nutritional stress during pregnancy in heifers [21].

The number of inseminations per conception was higher in heifers than in cows, while cattle subjected to synchronization protocols for the first postpartum AI required fewer inseminations than those inseminated after detected estrus. These findings are likely explained by lower pregnancy rate per AI and higher pregnancy loss in heifers, together with improved pregnancy outcomes associated with efficient synchronization protocols. Additionally, cattle experiencing early or late pregnancy loss required more inseminations per conception, as expected, because pregnancy loss may induce persistent uterine inflammation or delayed uterine involution, necessitating repeated breeding and additional reproductive treatments [35,36].

The likelihood of pregnancy within 360 days postpartum was influenced by the interval from calving to first AI, a history of pregnancy loss, and calving season. A longer interval to the first AI postpartum was associated with a lower likelihood of pregnancy and a longer calving-to-pregnancy interval, consistent with previous studies [37,38]. Although pregnancy rates per AI did not differ significantly by AI number, delaying the first AI postpones the start of breeding and shortens the available time to achieve conception, thereby lengthening the calving-to-pregnancy interval and reducing the probability of pregnancy within 360 days postpartum. A delayed first service may result from delayed resumption of ovarian cyclicity, incomplete uterine involution after calving, or failure to detect estrus. In this context, appropriate use of synchronization protocols, including PGF_2α_-GnRH, modified Double-Ovsynch, or modified Presynch-Ovsynch, may help shorten the interval from calving to first AI and thereby improve reproductive performance. Likewise, cows that experienced early or late pregnancy loss had a markedly reduced likelihood of pregnancy by 360 days postpartum and substantially longer calving-to-pregnancy intervals [39]. Pregnancy loss, irrespective of stage, may delay re-pregnancy because of an unfavorable uterine environment and the need for repeated insemination and reproductive treatments.

Calving season also influenced reproductive outcomes. Cows that calved in spring were more likely to conceive within 360 days postpartum than those that calved in winter, consistent with a previous study [8]. This effect may be related to peripartum nutritional and metabolic status. Hanwoo cattle are often fed similar rations year-round despite higher energy demands in winter; this practice may impair reproductive recovery in winter-calving cows. The observation that 80% of cows inseminated in spring had calved in winter helps explain why spring AI was associated with reduced pregnancy rates: many spring AI animals may have still been recovering from winter-related negative energy balance. By contrast, cows calving in spring may experience more favorable weather and feed availability, which can improve pregnancy outcomes.

Taken together, these findings identify clear, actionable targets for improving reproductive efficiency in Hanwoo herds. Priority interventions include strengthening peripartum nutrition and cold-stress mitigation for winter-calving cows; implementing heifer-specific measures, such as targeted nutritional support, enhanced estrus detection, and heifer-appropriate synchronization and AI timing; and, when appropriate, reducing the interval from calving to first AI by using timely synchronization protocols. Implementing these measures should increase pregnancy rates, reduce pregnancy loss, and shorten calving-to-pregnancy intervals at the herd level.

## CONCLUSION

Using large-scale field data, we identified modifiable drivers of reproductive performance in Hanwoo cattle. To improve outcomes, farms should strengthen peripartum nutrition and cold-stress mitigation for winter-calving cows inseminated in spring, tailor reproductive management to heifer physiology, and shorten time to first service by optimizing first AI timing and applying appropriate synchronization protocols, such as PGF_2α_-GnRH, modified Double-Ovsynch, or modified Presynch-Ovsynch. These measures can increase pregnancy rates, reduce pregnancy loss, and shorten calving-to-pregnancy intervals.

## Figures and Tables

**Table 1:** Overall reproductive performance of Hanwoo cattle

Category	Data from the present study		Data from previous studies	

	No. of cattle	Mean±SD or %	Mean1) or %	References
Heifers only				
Mean age at first AI in heifers (mo)	757	14.6±1.6	13.9	13
			14.8	14
			15.4	15
Mean age at first calving in heifers (mo)	657	25.2±2.5	24.9	14
			25.5	15
			27.0	16
Cows only				
Mean interval from calving to first postpartum AI in cows (d)	2,199	68.8±20.9	65.0	17
			69.5	15
			77.5	18
Mean interval from calving to pregnancy in cows (d)	2,144	91.2±51.0	72.1	17
			91.2	14
			100.5	19
Heifers and cows				
Pregnancy per AI at 59 d after insemination (%)	2,818/4,656	60.5%	61.5%	13
			62.4%	18
Mean number of inseminations per conception	2,780	1.53±0.86	1.33	16
			1.53	14
			1.54	19

1) Values reported in previous studies were not presented uniformly (for example, time units differed between d and mo, and variability was reported as either SD or standard error). Where possible, mean values were converted to common units and are shown here for ease of comparison; measures of variability were not consistently reported and therefore are not displayed.

SD, standard deviation; AI, artificial insemination.

**Table 2:** Factors affecting the probability of pregnancy per AI 59 d after first and subsequent inseminations in Hanwoo cattle

Parameter	Pregnancy per AI (n/n)1)	Odds ratio	95% CI	p-value2)
Season of AI				0.0166
Winter	63.0% (385/611)	Reference		
Spring	56.2% (592/1,054)	0.76	0.620–0.938	0.0102
Summer	62.0% (1,169/1,886)	0.96	0.790–1.157	0.6456
Autumn	60.8% (672/1,105)	0.92	0.747–1.125	0.4052
Parity				0.0093
Cow	62.0% (2,101/3,390)	Reference		
Heifer	56.6% (717/1,266)	0.84	0.729–0.957	0.0093
AI type3)				0.0040
IDE	57.8% (940/1,627)	Reference		
PGF_2α_-GnRH	62.4% (625/1,002)	1.19	1.011–1.399	0.0363
Ovsynch	56.2% (186/331)	0.91	0.717–1.162	0.4570
GnRH-Ovsynch	59.4% (266/448)	1.06	0.856–1.319	0.5826
Modified Double-Ovsynch	62.6% (585/935)	1.21	1.009–1.441	0.0395
Modified Presynch-Ovsynch	69.0% (216/313)	1.58	1.215–2.065	0.0007
AI number				0.0521
First AI	60.5% (1,804/2,981)	Reference		
Second AI	62.5% (698/1,116)	1.16	0.993–1.348	0.0613
≥Third AI	56.5% (316/559)	0.91	0.752–1.103	0.3393
Herd size4)				0.1749

1) n/n indicates the number of pregnancies confirmed at 59 d after AI divided by the number of inseminations evaluated for each category.

2) p-values indicate comparisons with the designated reference category within each model effect.

3) AI type: (1) IDE, AI after detected estrus; (2) PGF_2α_-GnRH, injection of 500 μg of a prostaglandin F_2α_ (PGF_2α_) analog followed by 10 μg of a gonadotropin-releasing hormone (GnRH) analog, with timed AI (TAI) 72 h after the PGF_2α_ injection; (3) Ovsynch, injection of GnRH followed by PGF_2α_ 7 d later, a second dose of GnRH 56 h after PGF_2α_, and TAI 16 h after the second GnRH injection; (4) GnRH-Ovsynch, injection of GnRH followed by the Ovsynch protocol 6 d later; (5) modified Double-Ovsynch, injection of GnRH followed by PGF_2α_ 11 d later, GnRH 3 d after the first PGF_2α_, and then the Ovsynch protocol 7 d later; and (6) modified Presynch-Ovsynch, injection of PGF_2α_ followed by a second dose of PGF_2α_ 11 d later, GnRH 3 d after the second PGF_2α_, and then the Ovsynch protocol 7 d later.

4) Herd size categorized according to the number of Hanwoo cattle (<60 or ≥60).

AI, artificial insemination; CI, confidence interval.

**Table 3:** Factors associated with early or late pregnancy loss1) in Hanwoo cattle

Parameter	Early pregnancy loss				Late pregnancy loss			

	Early loss % (n/n)2)	Odds ratio	95% CI	p-value	Late loss % (n/n)	Odds ratio	95% CI	p-value3)
Season of AI		-	-	0.4542		-	-	0.5454
Parity		-	-	0.4081		-	-	0.0225
Cow	1.8 (38/2,139)	-	-		1.8 (39/2,139)	Reference	-	
Heifer	2.2 (16/733)	-	-		3.3 (24/733)	1.82	1.088–3.052	0.0225
AI type		-	-	0.9974		-	-	0.2399
AI number				0.0394				0.5311
First AI	2.3 (43/1,847)	Reference			2.3 (42/1,847)	-	-	
Second AI	0.7 (5/703)	0.30	0.119–0.762	0.0113	2.0 (14/703)	-	-	
≥Third AI	1.9 (6/322)	0.80	0.336–1.887	0.6053	2.2 (7/322)	-	-	
Herd size4)				0.5505				0.9585

1) Early pregnancy loss is defined as loss occurring from 31 to 59 d of gestation; late pregnancy loss is defined as loss occurring from 60 to 260 d of gestation.

2) n/n indicates the number of losses divided by the number of pregnancies evaluated for each category.

3) p-values indicate comparisons with the designated reference category within each model effect.

4) Herd size categorized according to the number of Hanwoo cattle (<60 or ≥60).

AI, artificial insemination; CI, confidence interval.

**Table 4:** Least squares mean number of inseminations per conception in Hanwoo cattle

Parameter	Inseminations per conception	p-value1)
Parity		<0.0001
Cow (n = 2,062)	2.23±0.07	Reference
Heifer (n = 708)	2.39±0.07	<0.0001
Pregnancy loss category2)		<0.0001
No pregnancy loss (n = 2,698)	1.51±0.02	Reference
Early pregnancy loss (n = 42)	2.87±0.13	<0.0001
Late pregnancy loss (n = 30)	2.55±0.15	<0.0001
AI type for first AI3)		0.0101
IDE (n = 795)	2.40±0.07	Reference
PGF_2α_-GnRH (n = 474)	2.32±0.07	0.0910
Ovsynch (n = 107)	2.31±0.10	0.2937
GnRH-Ovsynch (n = 327)	2.35±0.08	0.3974
Modified Double Ovsynch (n = 817)	2.31±0.07	0.0420
Modified Presynch-Ovsynch (n = 250)	2.17±0.08	0.0002
Herd size4)		0.2737

1) p-values indicate comparisons with the designated reference category within each model effect.

2) Early pregnancy loss is defined as loss occurring from 31 to 59 d of gestation; late pregnancy loss is defined as loss occurring from 60 to 260 d of gestation.

3) AI type for first AI: (1) IDE, AI after detected estrus; (2) PGF_2α_-GnRH, injection of 500 μg of a prostaglandin F_2α_ (PGF_2α_) analog followed by 10 μg of a gonadotropin-releasing hormone (GnRH) analog, with timed AI (TAI) 72 h after the PGF_2α_ injection; (3) Ovsynch, injection of GnRH followed by PGF_2α_ 7 d later, a second dose of GnRH 56 h after PGF_2α_, and TAI 16 h after the second GnRH injection; (4) GnRH-Ovsynch, injection of GnRH followed by the Ovsynch protocol 6 d later; (5) modified Double-Ovsynch, injection of GnRH followed by PGF_2α_ 11 d later, GnRH 3 d after the first PGF_2α_, and then the Ovsynch protocol 7 d later; and (6) modified Presynch-Ovsynch, injection of PGF_2α_ followed by a second dose of PGF_2α_ 11 d later, GnRH 3 d after the second PGF_2α_, and then the Ovsynch protocol 7 d later.

4) Herd size categorized according to the number of Hanwoo cattle (<60 or ≥60).

AI, artificial insemination.

**Table 5:** Factors affecting the likelihood of pregnancy by 360 d postpartum in Hanwoo cows

Parameter	Hazard ratio	95% CI	p-value1)
Interval from calving to first AI			
≤60 d (n = 645)	Reference		
61 to 80 d (n = 1,093)	0.60	0.537–0.677	<0.0001
≥81 d (n = 406)	0.34	0.299–0.394	<0.0001
Pregnancy loss category2)			
No pregnancy loss (n = 2,087)	Reference		
Early pregnancy loss (n = 30)	0.28	0.190–0.408	<0.0001
Late pregnancy loss (n = 27)	0.08	0.044–0.144	<0.0001
Calving season			
Winter (n = 408)	Reference		
Spring (n = 921)	1.15	1.019–1.297	0.0237
Summer (n = 515)	1.06	0.926–1.213	0.4008
Autumn (n = 300)	1.02	0.878–1.195	0.7593
AI type for first AI3)			
IDE (n = 495)	Reference		
PGF_2α_-GnRH (n = 299)	0.94	0.812–1.095	0.4389
Ovsynch (n = 67)	0.97	0.744–1.274	0.8459
GnRH-Ovsynch (n = 272)	0.91	0.780–1.071	0.2667
Modified Double Ovsynch (n = 782)	0.97	0.848–1.102	0.6083
Modified Presynch-Ovsynch (n = 229)	1.09	0.912–1.291	0.3586
Herd size4)	-	-	0.2218

1) p-values indicate comparisons with the designated reference category within each model effect.

2) Early pregnancy loss is defined as loss occurring from 31 to 59 d of gestation; late pregnancy loss is defined as loss occurring from 60 to 260 d of gestation.

3) AI type for first AI: (1) IDE, AI after detected estrus; (2) PGF_2α_-GnRH, injection of 500 μg of a prostaglandin F_2α_ (PGF_2α_) analog followed by 10 μg of a gonadotropin-releasing hormone (GnRH) analog, with timed AI (TAI) 72 h after the PGF_2α_ injection; (3) Ovsynch, injection of GnRH followed by PGF_2α_ 7 d later, a second dose of GnRH 56 h after PGF_2α_, and TAI 16 h after the second GnRH injection; (4) GnRH-Ovsynch, injection of GnRH followed by the Ovsynch protocol 6 d later; (5) modified Double-Ovsynch, injection of GnRH followed by PGF_2α_ 11 d later, GnRH 3 d after the first PGF_2α_, and then the Ovsynch protocol 7 d later; and (6) modified Presynch-Ovsynch, injection of PGF_2α_ followed by a second dose of PGF_2α_ 11 d later, GnRH 3 d after the second PGF_2α_, and then the Ovsynch protocol 7 d later.

4) Herd size categorized according to the number of Hanwoo cattle (<60 or ≥60).

AI, artificial insemination; CI, confidence interval.

## SUPPLEMENTARY MATERIAL

**Supplement 1.** Distribution of insemination records (n=4,656) according to parity and model effects evaluated in Table 2.

**Supplement 2.** Distribution of confirmed pregnancies (n=2,872) according to parity and model effects evaluated in Table 3.

**Supplement 3.** Distribution of animals that conceived (n=2,770) according to parity and model effects evaluated in Table 4.

**Supplement 4.** Distribution of cows (n=2,144) according to model effects evaluated in Table 5.
