# Supplementary material for: Reproductive performance and its risk factors in Hanwoo cattle: a large-scale field study
Source: Anim Biosci. 2026 Jun 1;39(9):260249. doi: 10.5713/ab.260249 (PMC13547611; doi:10.5713/ab.260249)
Supplement: Supplementary file 1 [file ab-260249-Supplementary-1.pdf]

Supplement 1. Distribution of insemination records (n=4,656) according to parity and model effects evaluated in Table 2

Model effect	Category	Heifer (n)	Cow (n)	Total (n)
Season of AI	Winter	161	450	611
	Spring	296	758	1,054
	Summer	476	1,410	1,886
	Autumn	333	772	1,105
AI type	IDE	564	1,063	1,627
	$PGF_{2\alpha}$-GnRH	362	640	1,002
	Ovsynch	98	233	331
	GnRH-Ovsynch	103	345	448
	Modified Double-Ovsynch	91	844	935
	Modified Presynch-Ovsynch	48	265	313
AI number	First AI	758	2,223	2,981
	Second AI	314	802	1,116
	≥Third AI	194	365	559
Herd size	<60 cattle	618	1,644	2,262
	≥60 cattle	648	1,746	2,394
Total		1,266	3,390	4,656

Footnote: Values represent the number of insemination records (n) within each combination of parity and model effect category. IDE, insemination after detected estrus.
