# Supplementary material for: Reproductive performance and its risk factors in Hanwoo cattle: a large-scale field study
Source: Anim Biosci. 2026 Jun 1;39(9):260249. doi: 10.5713/ab.260249 (PMC13547611; doi:10.5713/ab.260249)
Supplement: Supplementary file 2 [file ab-260249-Supplementary-2.pdf]

Supplement 2. Distribution of confirmed pregnancies (n=2,872) according to parity and model effects evaluated in Table 3

Model effect	Category	Heifer (n)	Cow (n)	Total (n)
Season of AI	Winter	98	296	394
	Spring	166	434	600
	Summer	275	914	1,189
	Autumn	194	495	689
AI type	IDE	326	631	957
	$PGF_{2\alpha}$-GnRH	219	420	639
	Ovsynch	54	134	188
	GnRH-Ovsynch	52	214	266
	Modified Double-Ovsynch	51	549	600
	Modified Presynch-Ovsynch	31	191	222
AI number	First AI	434	1,413	1,847
	Second AI	196	507	703
	≥Third AI	103	219	322
Herd size	<60 cattle	361	1,062	1,423
	≥60 cattle	372	1,077	1,449
Total		733	2,139	2,872

Footnote: Values represent the number of confirmed pregnancies (n) within each combination of parity and model effect category. The dataset for Table 3 (n=2,872) comprises all pregnancies confirmed at the first ultrasound examination conducted at 31 days after AI, including those subsequently classified as maintained, early pregnancy loss (between 31 and 59 days of gestation), or late pregnancy loss (between 60 and 260 days of gestation). This differs from the denominator used in Table 2 (pregnancies maintained at 59 days after AI), which excludes early pregnancy losses.
