# Supplementary material for: Reproductive performance and its risk factors in Hanwoo cattle: a large-scale field study
Source: Anim Biosci. 2026 Jun 1;39(9):260249. doi: 10.5713/ab.260249 (PMC13547611; doi:10.5713/ab.260249)
Supplement: Supplementary file 3 [file ab-260249-Supplementary-3.pdf]

Supplement 3. Distribution of animals that conceived (n=2,770) according to parity and model effects evaluated in Table 4

Model effect	Category	Heifer (n)	Cow (n)	Total (n)
Pregnancy loss category	No pregnancy loss	678	2,020	2,698
	Early pregnancy loss	15	27	42
	Late pregnancy loss	15	15	30
AI type for first AI	IDE	316	479	795
	$PGF_{2\alpha}$-GnRH	181	293	474
	Ovsynch	41	66	107
	GnRH-Ovsynch	66	261	327
	Modified Double-Ovsynch	71	746	817
	Modified Presynch-Ovsynch	33	217	250
Herd size	<60 cattle	348	1,020	1,368
	≥60 cattle	360	1,042	1,402
Total		708	2,062	2,770

Footnote: Values represent the number of animals (n) that conceived during the study period, stratified by parity and model effect category. The analytical unit is the individual animal rather than the insemination event.
