# Supplementary material for: Reproductive performance and its risk factors in Hanwoo cattle: a large-scale field study
Source: Anim Biosci. 2026 Jun 1;39(9):260249. doi: 10.5713/ab.260249 (PMC13547611; doi:10.5713/ab.260249)
Supplement: Supplementary file 4 [file ab-260249-Supplementary-4.pdf]

Supplement 4. Distribution of cows (n=2,144) according to model effects evaluated in Table 5

Model effect	Category	n
Interval from calving to first AI	≤60 days	645
	61 to 80 days	1,093
	≥81 days	406
Pregnancy loss category	No pregnancy loss	2,087
	Early pregnancy loss	30
	Late pregnancy loss	27
Calving season	Winter	408
	Spring	921
	Summer	515
	Autumn	300
AI type for first AI	IDE	495
	$PGF_{2\alpha}$-GnRH	299
	Ovsynch	67
	GnRH-Ovsynch	272
	Modified Double-Ovsynch	782
	Modified Presynch-Ovsynch	229
Herd size	<60 cattle	1,068
	≥60 cattle	1,076
Total		2,144

Footnote: Values represent the number of cows (n) within each model effect category. The analytical unit is the individual cow. Heifers were not included in this analysis because the interval from calving to first AI is not applicable to nulliparous animals.
